# Wearable and flexible electrochemical sensors for sweat analysis: a review

**DOI:** 10.1038/s41378-022-00443-6

**Published:** 2023-01-01

**Authors:** Fupeng Gao, Chunxiu Liu, Lichao Zhang, Tiezhu Liu, Zheng Wang, Zixuan Song, Haoyuan Cai, Zhen Fang, Jiamin Chen, Junbo Wang, Mengdi Han, Jun Wang, Kai Lin, Ruoyong Wang, Mingxiao Li, Qian Mei, Xibo Ma, Shuli Liang, Guangyang Gou, Ning Xue

**Affiliations:** 1 School of Electronic, Electrical, and Communication Engineering, University of Chinese Academy of Sciences (UCAS), 100190 Beijing, China; 2 State Key Laboratory of Transducer Technology, Aerospace Information Research Institute (AIR), Chinese Academy of Sciences, 100190 Beijing, China; 3 Department of Biomedical Engineering, College of Future Technology, Peking University, 100871 Beijing, China; 4 Beijing Shuimujiheng Biotechnology Company, 101102 Beijing, China; 5 PLA Air Force Characteristic Medical Center, 100142 Beijing, China; 6 Institute of Microelectronics of the Chinese Academy of Sciences, 100029 Beijing, China; 7 CAS Key Laboratory of Biomedical Diagnostics, Suzhou Institute of Biomedical Engineering and Technology, Chinese Academy of Sciences (CAS), 215163 Suzhou, China; 8 CBSR&NLPR, Institute of Automation, Chinese Academy of Sciences, Beijing, China; 9 Functional Neurosurgery Department, Beijing Children’s Hospital, Capital Medical University, 100045 Beijing, China

**Keywords:** Chemistry, Nanoscale materials, Nanobiotechnology

## Abstract

Flexible wearable sweat sensors allow continuous, real-time, noninvasive detection of sweat analytes, provide insight into human physiology at the molecular level, and have received significant attention for their promising applications in personalized health monitoring. Electrochemical sensors are the best choice for wearable sweat sensors due to their high performance, low cost, miniaturization, and wide applicability. Recent developments in soft microfluidics, multiplexed biosensing, energy harvesting devices, and materials have advanced the compatibility of wearable electrochemical sweat-sensing platforms. In this review, we summarize the potential of sweat for medical detection and methods for sweat stimulation and collection. This paper provides an overview of the components of wearable sweat sensors and recent developments in materials and power supply technologies and highlights some typical sensing platforms for different types of analytes. Finally, the paper ends with a discussion of the challenges and a view of the prospective development of this exciting field.

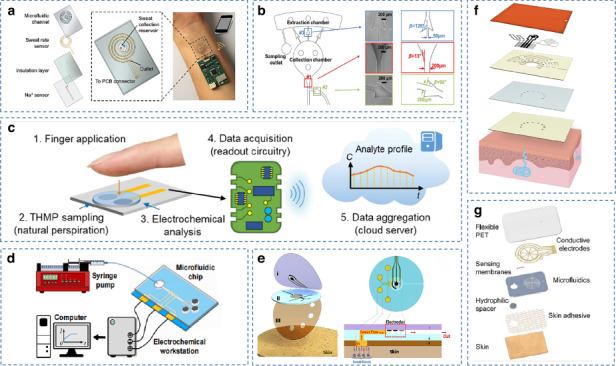

## Introduction

The ideal health care system would provide health condition monitoring and treatment before the onset of the disease. When a patient shows suboptimal health, the system would be able to detect it and address it. Currently, patients usually contact doctors for medical treatment after they have developed ailments with noticeable symptoms and thereafter receive passive treatment and monitoring by specialists. Therefore, there is an urgent need for a means to enable individuals to monitor themselves for early detection and timely management of disease without the involvement of expensive equipment or trained professionals.

With the development of medical diagnostic technology, the field of wearable biosensors is growing, and it offers innovative solutions to current medical problems^1^. Wearable biosensors are able to provide continuous, real-time physiological information via dynamic, noninvasive measurements of biochemical markers in biofluids while directly in contact with the sampled biofluids without inducing discomfort to the wearer^2^. In recent years, there has been a rapid development of wearable electronic devices that can accurately measure vital signs such as heart rate, body temperature, and blood pressure, helping to describe and monitor personal health conditions^3–6^. However, these biophysical parameters lack direct information on the dynamic biochemical and metabolic processes of the human body^7^. Biofluids (such as sweat, tears, saliva, or tissue fluids) are of interest as analytes for their ease of sampling and have shown the potential to provide continuous, real-time physiological information by understanding the body’s deeper biomolecular state^8,9^.

Compared to other biofluids, sweat is rich in analytes that may convey physiological information about the body and are closely correlated with blood levels, offering tremendous advantages in wearable sensing. Wearable sweat monitoring platforms allow sweat collection and analysis at the location of sweat production, resulting in autonomous, continuous, real-time sensing. In situ quantitative analysis of sweat is important for monitoring physiological health status and diagnosing diseases^10^. Since the first proposal of wearable sweat sensors for real-time analysis of sweat lactate in 2013^11^, many successors have achieved the monitoring of electrolytes, metabolites, drugs, trace elements, etc., in sweat. Optical methods such as fluorescence sensing and colorimetric methods, as well as electrochemical sensing methods, have emerged in the detection of sweat analytes. Electrochemical sensing is a common and well-established method for sweat analysis that is widely used in wearable sensors^12^ and dominates clinical diagnostics because of its high performance, portability, simplicity, and low cost^13,14^.

Some recent technological developments are driving the growing sophistication of wearable electrochemical sweat sensors. The fully integrated multiplexed sensing system for the simultaneous detection of multiple analytes makes sweat sensors more practical, providing a versatile wearable platform for large-scale clinical and physiological studies^7,15^. The application of iontophoresis has made it possible to actively induce sweat in sedentary scenarios, thus meeting the requirements for disease diagnosis and health monitoring^16,17^. The integration of microfluidic technology overcomes many issues that diminish data integrity and enhances sweat collection while greatly improving sample transport for better temporal resolution and accuracy in sweat analyte measurements^12,18^. In terms of wearable energy systems, the development of self-powered wearable sensors that integrate energy harvesting devices and energy storage devices facilitates the design and operation of efficient, sustainable, and autonomous wearable systems^19,20^. Moreover, these sensors will generate a large amount of time-series data, which can be analyzed through big-data techniques, promoting the advent of a personalized and intelligent era in medicine.

This review provides a comprehensive summary of recent advances in wearable electrochemical sweat sensors. First, we summarize the advantages of sweat for wearable sweat sensing, present in detail the various analytes in sweat that reflect the physiological information of the human body, and highlight the methods of sweat stimulation and collection. Second, we show the components of wearable electrochemical sweat sensors. For sensing elements, it is necessary to consider the selection of appropriate sensing modes and electrochemical detection methods for different analytes. For electronic components, in addition to the realization of functions such as signal processing and wireless data transmission, the optimization of the power supply method needs to be emphasized. Third, we present in detail some typical sensing devices that are significant in the development history of wearable electrochemical sweat sensors for different analytes. Finally, we summarize the future challenges and possible directions for the development of wearable sweat sensors. Wearable electrochemical sweat sensors have great advantages and potential for biomedical sensing.

## Sweat

### The advantages of sweat as a sample for analysis

The rapid development of wearable chemical sensors allows the noninvasive detection of analytes in accessible biofluids, providing a window into the overall dynamic biomolecular state of the human body. There are several candidate biofluids, but most of them have limitations in wearable sensing.

Blood is considered the gold standard for medical monitoring of analytes^3,21^. However, invasive methods of blood sampling pose a major obstacle for patients, especially newborns, elderly individuals, and hemophobic patients, for whom blood sampling can be challenging. Urine is also commonly used as a clinical medical sample but is not suitable for autonomous and continuous monitoring^22^. Tears contain certain salts, enzymes, proteins, and lipids that can reveal conditions and diseases of the eyes^23^. However, the existing tear sample collection protocols can cause eye irritation and produce reflex tears, which can affect the sensor’s test results^13^. Saliva contains a variety of biomarkers, including hormones, enzymes, antibodies, and antimicrobial agents, that can accurately reflect human status^24^. Unfortunately, however, saliva monitoring is also a challenge, as the mouth may contain many impurities, such as food particles, that affect the reliability of the data. The concentration of analytes in tissue fluids is very similar to that in blood^15^. However, it is necessary to use a fine needle or subcutaneous excitation current to collect samples, which can irritate the dermal tissue and cause discomfort^25^.

In contrast to other biofluids, sweat has tremendous advantages in wearable sensing. Sweat regulates the body’s heat balance^26^ and plays an important physiological role in thermoregulation, moisturization, immune defense, and electrolyte and pH balance^27,28^. It contains a wealth of substances that could potentially convey physiological information about the body^8,29–31^ and can indicate the health status of the body at the molecular level^22,32^. In addition, sweat is secreted by sweat glands that are widely distributed across the entire body. Thus, sweat can be obtained in a noninvasive manner at suitable locations on the body, ideal for continuous monitoring. Sweat sensors can be placed close to locations of sweat generation, allowing for rapid detection before analytes biodegrade. Some analytes in the blood, such as glucose^33^, lactate^34^, and ethanol^35^, have been reported to correlate strongly with the levels of chemical molecules in sweat. In a way, blood analysis can be replaced by sweat analysis. Although sweat measurement still has some issues with data reliability and the secretion mechanism of some analytes is still unknown, the advantages of sweat over other biofluids are rapidly bringing it to the forefront of wearable technology innovation^36^.

### Biomarkers in sweat

Sweat is rich in biomarkers: electrolytes (e.g., sodium, potassium, chloride, ammonium, calcium), metabolites (e.g., glucose, lactate, alcohol), trace elements (e.g., iron, zinc, copper), small molecules (e.g., cortisol, urea, tyrosine), neuropeptides and cytokines^8,22,30^. As sweat contains a wealth of physiological information, wearable sweat sensors have a wide range of promising applications in areas such as fitness tracking and health monitoring in high-performance sports for athletes, as well as disease diagnosis and medical monitoring^1,21^ (Table 1).

**Table 1:** Key analytes in sweat and associated health conditions

Analyte category	Analyte	Health condition	Ref.
Electrolytes	Na^+^	Dehydration, hyponatremia, electrolyte imbalance	^40–43,45,47^
	Cl^-^	Dehydration, cystic fibrosis	^17,50,51^
	K^+^	Hypokalemia, muscle cramps	^52,53^
	Ca^2+^	Myeloma, cirrhosis, renal failure, acid–base balance disorder	^54^
	NH^4+^	Shift from aerobic to anaerobic metabolic conditions	^9,41,55^
	PH	Pathogenesis of skin diseases, wound healing	^30,38,43,143^
Metabolites	Glucose	Diabetes	^17,33,52,103^
	Lactate	Shift from aerobic to anaerobic metabolic conditions	^7,11,58,37^
	Alcohol	Inebriation	^16,76,77^
	Uric acid	Renal dysfunction, gout	^61,144^
Drugs	Caffeine	Coronary syndrome, hypertension, Depression	^63,145^
	Levodopa	Parkinson’s disease	^64,89^
Trace metals	Zn^2+^	Stress and immune system-induced muscle damage	^44,66,67^
	Cu^2+^	Rheumatoid arthritis, Wilson’s disease, cirrhosis of the liver	^44,66,68^
Other analytes (hormones, cytokines, proteins, etc.)	Interleukin 6	Insulin activity, immune responses in cancer therapy	^69,70,145^
	Cortisol	Stress	^59,69,72,73^
	Tyrosine	Metabolic disorders, tyrosinemia	^74^
	Neuropeptide Y	Stress	^30,75^

### Electrolytes

Water molecules are the most common component of sweat, making up ~99% of its contents^37^. Sweat is slightly acidic, with an average pH of 6.3, which is more acidic than blood^38^. PH measurements can reflect changes in the concentration of various electrolytes in sweat, thus conveying information about the disease and metabolic activity^30^. In addition, local monitoring of pH helps in wound healing, detection of infection, and treatment for metabolic alkalosis^39^. Na^+^ and Cl^−^ are the most abundant electrolytes in human sweat^40^ and are the driving force for the entry of water into a sweat, which can be used to assess the magnitude of the sweating rate^41^. At the same time, Na^+^ is an important biomarker of electrolyte imbalance in the human body and is essential for the regulation of osmolality, water balance, and pH^42^, especially for athletes who exercise for long periods or those exposed to heat and humidity^43–49^. Chloride analysis in sweat has been used as the gold standard for the diagnosis of cystic fibrosis^50^. Sweat collection and detection methods by iontophoresis can provide rapid screening for cystic fibrosis disease^17,51^. The concentration of potassium in sweat is proportional to that of potassium in the blood. Low levels of potassium in sweat may indicate dehydration, which, in addition to causing muscle cramps, may be life-threatening in patients with cardiovascular disease^52,53^. Monitoring Ca^2+^ levels in sweat can predict diseases such as myeloma, acid–base balance disorder, cirrhosis, renal failure, and normocalciuric hyperparathyroidism^54^. Ammonium levels in sweat are directly related to plasma ammonium concentrations. Ammonia secretion in sweat can be used as an indicator of protein metabolic breakdown and can be used to monitor changes during the transition from aerobic to anaerobic exercise^9,41,55^.

### Metabolites

Glucose in sweat is a representative molecule and is a typical indicator for studying sweat secretion pathways, sweat production rates, and dilution of analyte concentrations. It has been reported that iontophoresis-induced glucose levels in sweat may correlate with glucose levels in the blood, making it suitable for diabetes screening and monitoring^3,33^. Apart from this, a recent study demonstrated that the use of glucose-loaded liposomes (GLLs) can be used for competitive colorimetric immunoassays of small-molecule antibiotics such as streptomycin (STR)^56^. The concentration of lactate in sweat is very close to that in blood and can be used as a sensitive marker of tissue viability and may provide a warning for stress ischemia^11,57^. It has also been shown that the sweat secretion rate and lactate content are independent of each other and are very suitable for noninvasive diagnosis^58^. Studies have shown that sweat ethanol concentration is highly correlated with blood ethanol concentration, which allows continuous noninvasive blood alcohol monitoring by monitoring sweat alcohol concentration^16,35^. Uric acid (UA) levels in sweat can provide insight into kidney disease^59^. It has been reported that UA levels in the sweat of patients with gout are higher than those of healthy individuals, and UA has been widely used in the clinical treatment of gout^60,61^.

### Drugs and trace metals

As the body excretes toxins, so can drugs be secreted in the sweat^62^. Chronic caffeine overdose can lead to health problems such as coronary syndrome, hypertension, and depression. Caffeine concentrations in sweat have been reported to correlate with plasma and urine caffeine concentrations^63^. The concentration of levodopa in sweat is correlated with that in plasma. The monitoring of levodopa in sweat can provide a basis for dose optimization^64^.

Sweat is one of the most important routes for the detoxification of heavy metals^65^. Perspiration represents an important pathway for the secretion of zinc. Trends in zinc concentration can be used as an indicator to monitor the body for stress and immune system-induced muscle damage^66,67^. It is extremely important to determine the loss of zinc during physical activity by monitoring zinc in sweat. Serum copper is used as an indicator to detect rheumatoid arthritis, Wilson’s disease, and cirrhosis of the liver. In addition, the levels of copper and other heavy metals in sweat can reflect physical exercise, heat stress, and diet^66,68^.

### Other analytes

In addition to the ions and molecules mentioned above, there are also hormone-like molecules and small molecule proteins in sweat, such as cortisol, neuropeptides, and cytokines^4^. Interleukin 6 (IL-6) is an inflammatory pluripotent stem cell factor consisting of 212 amino acids^69^ that affects insulin activity and has the potential to be used to monitor immune responses in cancer therapy^70^. A recent study showed that IL-6 is released upon infection with SARS-CoV-2 (causing COVID-19) and stimulates inflammatory pathways as part of the acute phase response^71^. Cortisol is a steroid hormone that is released by the body in response to psychological and physical stress and therefore plays an important role in the body’s stress response, regulation of metabolism, and immune response^72,73^. Tyrosine is a conditionally essential amino acid^61^ that is closely related to metabolism, and abnormal tyrosine concentrations can indicate metabolic disorders such as tyrosinemia^74^. Neuropeptide Y (NPY) is involved in the body’s stress response and is found in higher concentrations in the sweat of patients with depression than in that of people without depression^75^.

### Sweat stimulation

Sweat samples can be obtained by two approaches: the passive approach and the active approach. In the passive approach, people intensely exercise, such as by running, cycling, or performing other physical exercises, to induce sufficient sweat secretion. In the active approach, people are subjected to electrical stimulation.

**Fig. 1:**
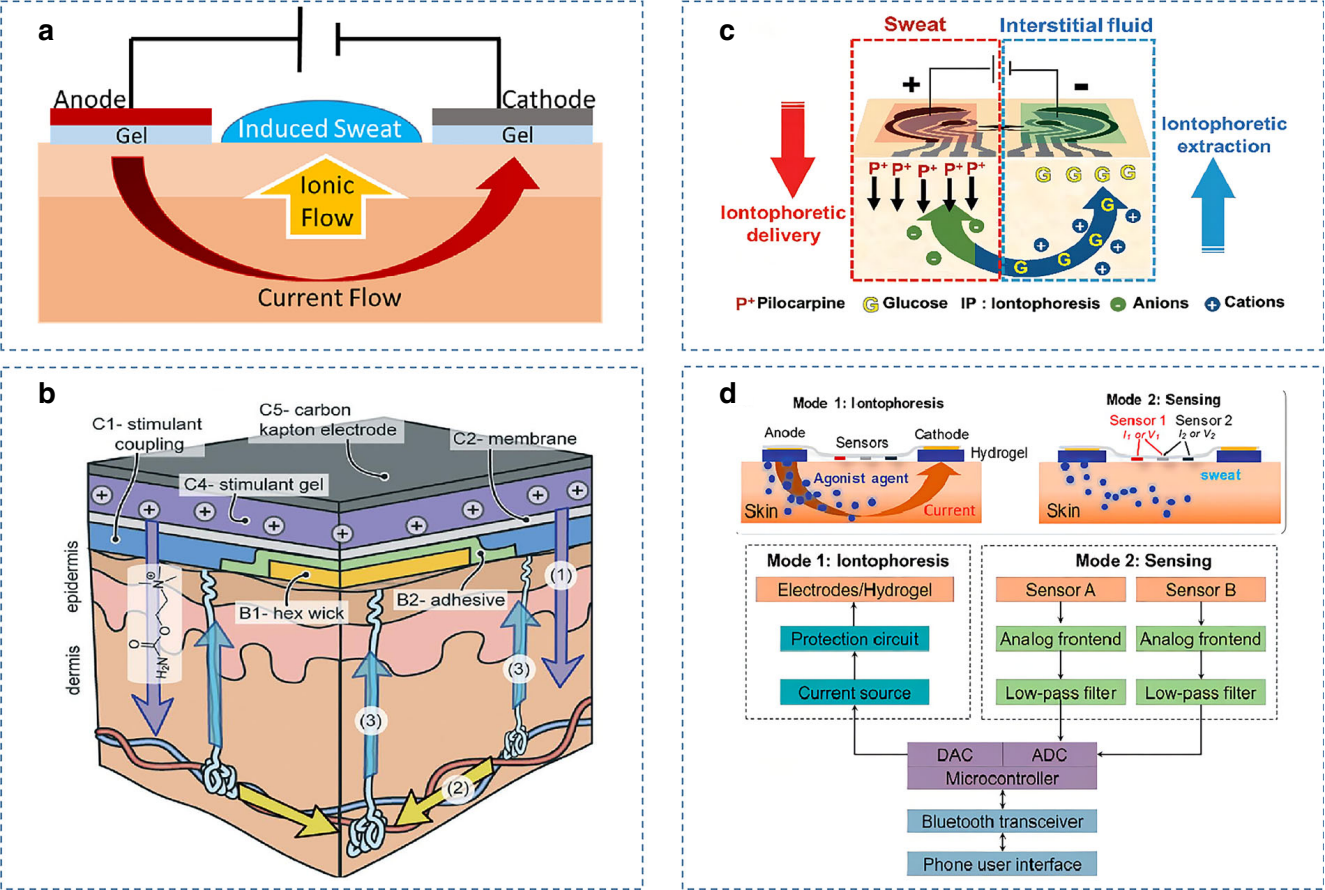
Examples of wearable sweat sensors based on iontophoresis. **a** The basic principle of iontophoresis^21^. Copyright 2019, Elsevier. **b** An improved sweat alcohol sensor based on iontophoresis using a hex-wick^35^. Copyright 2018, RSC. **c** An iontophoresis-based biosensor for dual biofluid sampling and analysis, simultaneously and actively stimulating the production of sweat and tissue fluids^77^. Copyright 2018, Wiley. **d** A fully integrated and autonomous ionophoresis-based sweat sensor with a programmable current source^17^. Copyright 2017, National Academy of Sciences USA

Iontophoresis is a widely used method of active sweat induction, allowing the acquisition of sweat samples while the body is sedentary^22^. As shown in Fig. 1a, a current is generated under the skin surface by applying a voltage between the iontophoretic electrodes, allowing the agonist (e.g., pilocarpine molecules) to be delivered to the sweat gland at the anode and stimulating the secretion of sweat. This method has been applied to monitor levels of chloride^17^, ethanol^16,76,77^, and glucose^78,79^.

A wearable sweat alcohol biosensing device based on iontophoresis achieved further innovations in maintaining the data integrity of sweat samples^35^. As shown in Fig. 1b, the device isolates sweat irritants from the skin with a membrane that effectively prevents sweat from diluting the irritants. Sudomotor axon reflex sweating is used to help minimize mixing between fresh sweat and old, contaminated sweat. The use of carbachol as an agonist facilitates a longer and more stable rate of sweat production. In addition, the “hex wick” material is used in the coupling of sweat to the sensor, which can effectively prevent skin contamination and transport the sample to the sensor quickly, reducing the latency time caused by fluid sampling.

Compared to single biofluid monitoring, simultaneous analysis of sweat and ISF could expand the scope of detecting biomarkers and improve clinical accuracy. Figure 1c shows a study demonstrating the possibility of a dual epidermal fluid sampling system^80^. This biosensing system is achieved by the parallel operation of reverse iontophoresis ISF extraction from the skin in the cathodic chamber and ionophoresis in the anodic chamber to supply a sweat-inducing drug (pilocarpine) into the skin at different locations. It successfully achieved the simultaneous detection of alcohol in sweat and glucose in ISF. This parallel sweat and ISF monitoring combine the advantages of both to expand the range of detectable biomarkers and enhance the accuracy of the detection results. In the future, this approach to biofluid stimulation could be extended to other biomarkers in sweat and ISF for additional medical applications.

However, the conventional iontophoresis technique tends to corrode the electrodes and cause strong irritation, causing skin discomfort. To address this problem, a programmable current source is used to periodically induce sweat and an upper limit of the ion electroosmotic current is set as a safety mechanism, which can effectively avoid overheating and burning of the skin (Fig. 1d)^17^.

### Sweat collection and microfluidic devices

There are two ways to collect sweat samples. One way is to use disposable gauze, absorbent pads, arm bags, and gloves to extract sweat^81^. Some commercial sweat collection devices direct sweat produced by the skin into a sealed chamber. For example, the Macroduct collection system can induce sweat by Pilogel^®^ Iontophoretic Disks and detect chloride in sweat to compare sweat tests used in diagnosing cystic fibrosis (CF)^10,82^. The drawback of this instrument is that an additional device is required for sweat analysis; thus, it cannot realize in situ sweat analysis.

**Fig. 2:**
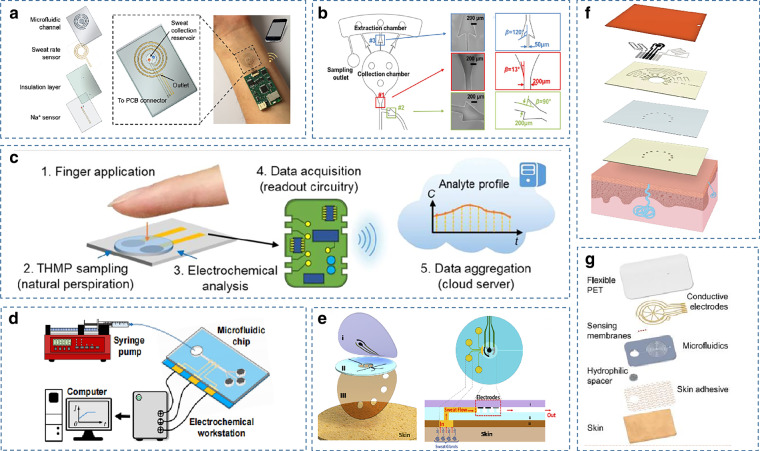
Examples of microfluidic sweat sensors. **a** The structure of a capillary force-driven microfluidic sweat sensor^82^. Copyright 2018, ACS. **b** A thin, soft, skin-mounted microfluidic network with capillary bursting valves^145^. Copyright 2017, Wiley. **c** A hydrogel-based microfluidic sweat sensor with natural permeation sampling for wireless chemical/biological sensing^78^. Copyright 2019, ACS. **d** An evaporative micropump-driven electrochemical sensor^79^. Copyright 2019, MDPI. **e** A microfluidic electrochemical detection system with enhanced sweat sampling and metabolite detection^18^. Copyright 2017, ACS. **f** A microfluidic system based on laser engraving^61^. Copyright 2020, Springer Nature. **g** A microfluidic sweat sensor for continuous analysis of body thermoregulatory sweat at rest^82^. Copyright 2021, Springer Nature

Compared with blood or other body fluids, the content of biomarkers in sweat is lower, which is not conducive to reliable and accurate real-time monitoring of sweat. Addressing this challenge requires significant advances in sweat collection and transport. One promising method is to channel the generated sweat through a microfluidic tube into a reservoir for sample storage and analysis. Sweat collection can be enhanced by minimizing sample leakage, evaporation, and contamination, improving the temporal resolution and accuracy of sweat analyte measurements. Microfluidic devices require a driving force to ensure the continuity of sweat delivery. In addition to using the sweat glands themselves as a pressure source to drive the fluid, capillary force, osmotic pressure, and evaporation pumps can all activate sweat delivery without the need for complex external equipment^12^. Capillary force promotes the flow of sweat along microfluidic channels (Fig. 2a)^83^. By adding a valve structure or hydrophilic treatment of the channel surface, the direction of sweat flow can be further controlled (Fig. 2b)^84^. To achieve a shorter sampling time and higher sampling efficiency, a hydrogel can typically be placed at the inlet of the microfluidic chip. There is an osmotic pressure difference between the hydrogel and the sweat, thus fluid is pumped into the microchannels (Fig. 2c)^85^. Furthermore, by designing micropores at the outlet of the microfluidic chip, an evaporation-driven micropump can effectively achieve a continuous flow of sweat. The flow rate can be easily controlled by changing the number or shape of microperforations (Fig. 2d)^86^.

Joseph Wang’s group further extended the principle of microfluidic sweat sampling by integrating an electrochemical biosensor into the reservoir of the microfluidic device for the first time, enabling real-time continuous monitoring of sweat metabolites such as glucose and lactate^18^. The device combines the advantages of electrochemical epidermal sensing and the microfluidic device. A short sweat sampling time, fast sweat flow rate, and efficient transmission over the detector surface are achieved, and the problems of sweat mixing and skin-worn chemical sensors are solved. A key bottleneck in sweat analysis using microfluidics is to achieve uniform, high-throughput manufacturing of sweat sensor components. To overcome this challenge, a microfluidic sensing patch manufactured on a large scale by introducing a roll-to-roll (R2R) process allows real-time measurement of Na^+^, K^+^, and glucose in sweat. Furthermore, most of the reported wearable microfluidic platforms are based on silicon elastomers, which require complex manufacturing processes and expensive microfabrication equipment^87^. One promising manufacturing technology is CO_2_ laser engraving, which allows for rapid engraving of patterns under ambient conditions and reduces personnel training and process optimization. Figure 2f shows an entirely laser-engraved sweat sensor. It is reported to allow wireless continuous monitoring of uric acid (UA), tyrosine (Tyr), and vital signs in sweat. The sensing platform employs two laser engraving modes: a grating mode for chemical sensors and a vector mode for microfluidic patterns as well as physical sensors. This multimode microfluidic sensor patch can be fabricated on a large scale, and it has the ability to achieve efficient microfluidic sweat sampling, sensitive molecular sensing, and multiplexed vital sign sensing^61^.

However, in most cases, strenuous exercise or pharmacological stimulation is required to produce sufficient quantities of sweat for analysis. The low secretion rate and rapid evaporation of sweat at rest limit the volume available to be collected in a sensor. For patients who are unable to exercise or who are sensitive to the burn of the skin by iontophoresis, a sweat collection method that uses a waterproof, skin-contact microfluidic system that can capture the natural release of sweat is a good solution^88^. However, this method can only capture sweat after warm water showering or bathing and cannot continuously analyze thermoregulatory sweat at rest. Recently, a microfluidic-based sweat patch has been successfully developed to achieve continuous measurement of the at-rest thermoregulatory sweat composition and rate. As shown in Fig. 2g, the device consists of three parts: a microfluidic layer, an electrochemical sensing electrode, and a laminated hydrophilic housing. The sweat collection wells of the microfluidic layer are filled with SU8 padding coated with hydrogel, which can quickly absorb sweat at a low secretion rate, effectively solving the problem of dead volume^89^. This wearable sweat sensor fills a gap in the field of devices for the continuous detection of resting sweat and helps better study correlations between sweating rates and sweat composition.

### Electrochemical sweat sensor components

Wearable electrochemical sweat sensing systems require precise measurement of biomarkers in sweat, with different sensing modalities depending on the characteristics of the target analytes. To meet the needs for lightweight, small size, portability, and comfortable attachment to the skin, the sensor material needs to be flexible and stretchable. At the same time, electronic modules and wireless communication strategies need to be integrated to realize real-time sweat analysis, and power consumption needs to be considered. In this section, we introduce these elements.

### Electrochemical detection methods

Electrochemical detection methods have the advantages of high sensitivity, high selectivity, fast response time, and simple wearable design. Currently, most electrochemical sweat sensing devices base their measurements on amperometry, potentiometry, and voltammetry^4^. The main considerations in the selection of the sensing method are the characteristics of the target biomarker in sweat, especially its molecular properties and concentration range^21^. In addition to the detection methods mentioned above, there are also gas-based contactless electrochemical biosensors. Compared with traditional electrochemical biosensors, gas-based contactless sensing interfaces can be separated from the bioassembly platform, effectively avoiding the complex process of biological assembly on the sensing interface, thus having better reproducibility and stability^90^. It is worth noting that photoelectrochemical detection, which is an important branch of electrochemical detection, has superior sensing performance and has become a research hot spot in analytical chemistry in the past few years^91^. Although we have not seen any reports on sweat sensors using photoelectrochemical detection methods, combining photoelectrochemical detection with sweat analyte detection is extremely promising in the future.

Potentiometry was used to determine the changes in ion concentration in a solution by measuring the potential between the sensing electrode and the reference electrode. By this method, selective detection of Na^+7,^^42^, K^+7,^^52^, Ca^2+,^^54^, NH_4_^+^^11^, and pH^92^ in sweat solutions has been successfully achieved. In addition, potentiometry has been developed for the detection of protein-specific immunosensors. The output signal can be effectively amplified by modifying oligonucleotides on gold nanoparticles. Amperometric sensors have a three-electrode structure that includes a working electrode where the analyte reacts, a fixed-potential electrode that serves as a reference for the working electrode, and a counter electrode used as a collector. For example, glucose^93^, lactate^11^, and alcohol^16,35^ can be detected by modifying selective enzymes on the electrode surface and detecting the current generated by the redox reaction of the analyte with the enzyme. The voltammetric sensor also has a three-electrode structure, where a voltage scan is applied between the working and reference electrodes to cause a redox reaction with the target analyte. By measuring the peak redox current, the concentration of the target analyte is deduced. Unlike potentiometry or amperometry, voltammetry can measure multiple analytes simultaneously. There are three main types of voltammetry: square wave solvation voltammetry (SWASV), cyclic voltammetry (CV), and differential pulse voltammetry (DPV). SWASV is generally used to detect trace metals in sweat, such as copper, zinc, and gold^66–68^. CV and DPV are suitable for drug detection in sweat, for example, for caffeine^63^ by DPV and levodopa^64^ by CV.

### Material selection

**Fig. 3:**
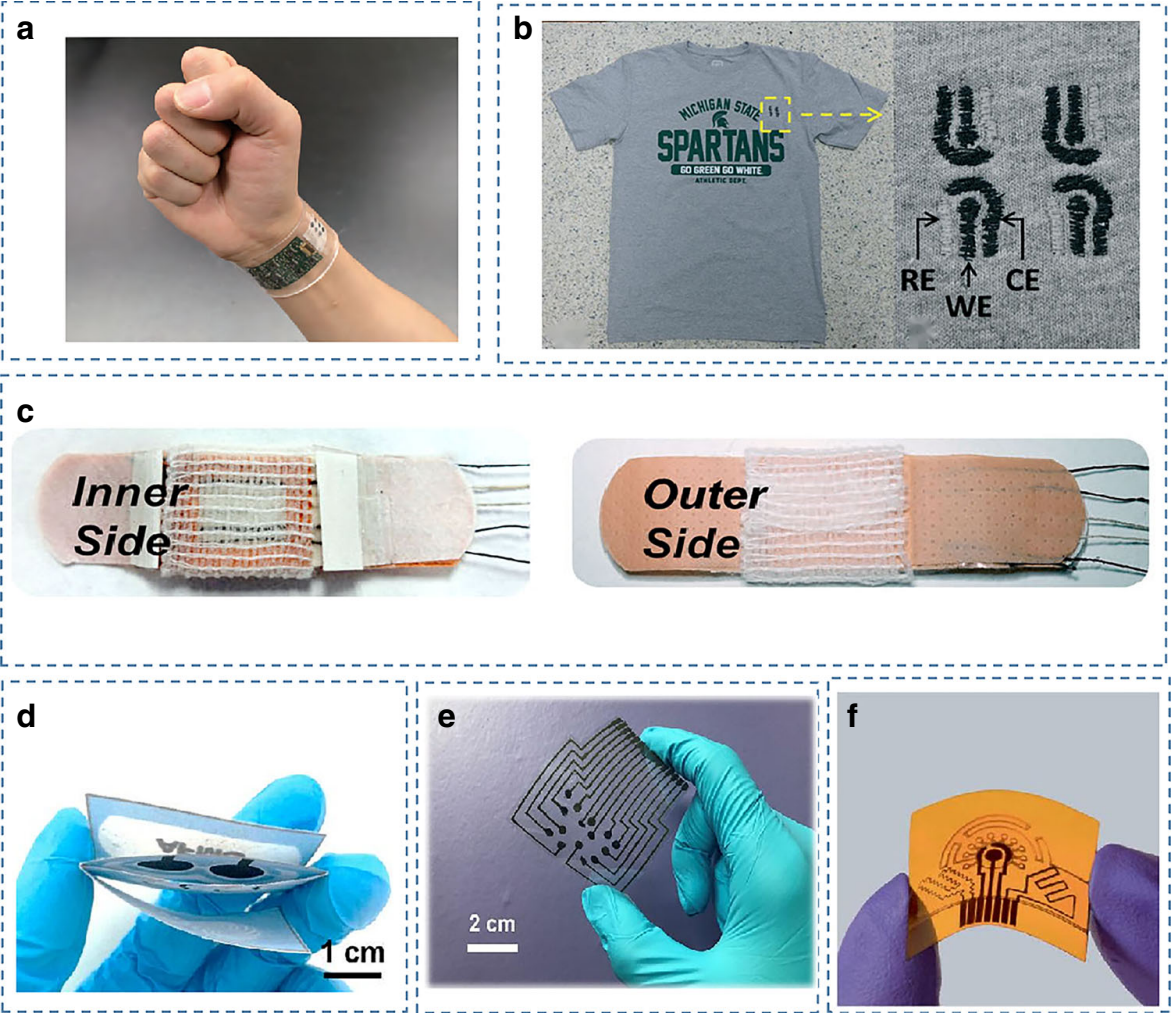
Examples of wearable sweat sensors made of different materials. **a** A fully integrated wearable sensing platform using PET as the substrate^7^. Copyright 2016, Springer Nature. **b** An embroidered electrochemical sensor for biomolecular detection fabricated on a cotton t-shirt^104^. Copyright 2016, RSC. **c** A thread-based multiplexed sensor patch for sweat monitoring^9^. Copyright 2020, Springer Nature. **d** A sweat analysis patch using a foldable all-paper substrate^31^. Copyright 2021, Elsevier. **e** A wearable electrochemical sensing array using SilkNCT as the working electrode^117^. Copyright 2019, AAAS. **f** A wearable laser-engraved sensor for sweat detection^61^. Copyright 2020, Springer Nature

Wearable devices are in direct contact with the skin and require the ability to detect the wearer’s physiological information continuously and stably, minimizing interference with the wearer’s daily life. Therefore, the basic material requirement for wearable sweat sensors is to conform to the geometry of the skin, which needs to satisfy flexibility and stretchability while maintaining ideal electrochemical and mechanical stability. Materials with relatively low Young’s modulus are mostly selected as substrates, and currently, the commonly used materials for flexible sensors are fabric, paper, flexible polymers, and plastics^94,95^ (Fig. 3).

Conventional flexible substrates are currently composed mainly of polymers such as polyethylene terephthalate (PET)^7^, polyimide (PI)^96^, polydimethylsiloxane (PDMS)^97–99^, polyurethane (PU)^100^, and polymethyl methacrylate (PMMA)^101^. These polymer films have excellent properties suitable for wearable devices, such as low cost, good biocompatibility, excellent physical–mechanical properties, and electrical insulation properties. The microstructure of the device is equally important to improve the sensitivity and response speed of flexible pressure sensors. It has been reported that a flexible pressure sensor with a saw-toothed microstructure made of two crossed PDMS films enables simple and fast detection of target concentrations^102^. However, the mismatch between these substrates and the epidermis leads to sweat accumulation, making the test results inaccurate. they also have poor breathability and stretchability, making them usable on the skin for only a short period.

In contrast, textile-based platforms provide natural breathability for the underlying skin, promoting natural sweating and evaporative cooling^9^, and they have high flexibility, softness, and comfort^103^. Moreover, the textile-based approach can be integrated into existing garments such as socks^19^ and T-shirts^104^, making sweat monitoring more versatile. Fabric-based wearable sensors are currently fabricated in two main ways. One approach is to print fully formed sensors onto fabric via a screen printing process, which can provide mass production of low-cost reproducible textile sensors^53^. Another approach is to form thread-based sensors that can be processed into the fabrics themselves. For example, a smart textile electrochemical sweat lactate sensing device was reported, assembled by weaving three different functional stretchable gold fiber electrodes into the fabric to monitor the lactate concentration in sweat, maintaining high performance even under high tensile strain^105^. Advances in textile-based sensors have offered considerable advantages in the field of on-body monitoring devices, as they conform with the wearer’s anatomy while enabling unobtrusive sensing. However, the implementation of fabric-based electrochemical sensors relies on screen printing or applying coatings to fabrics, leading to inhomogeneity and variability due to the core wicking/diffusion of inks on unevenly textured fabric surfaces. Recently, to address this issue, a bottom-up approach was proposed that used individual functionalized threads woven together as a substrate and encapsulated as a patch to form a wearable multiplexed sweat-sensing platform^9^.

Paper is also an ideal substrate material with excellent properties, such as low cost, easy preparation, good biocompatibility, and the inherent wicking ability of hydrophilicity. Through hydrophilic and hydrophobic material modification, wax dyeing, inkjet printing, and photolithography, self-absorbing sweat microfluidic channels can be generated in paper-based devices relatively easily. At the same time, the softness and foldability of paper facilitate the reduction of device size and the construction of devices with multiple functions^31^. For example, Tang et al. used paper as an electrode to fabricate a flexible pressure sensor for point-of-care immunoassays, significantly improving the sensitivity of target detection^106^. Apart from this, paper-based analytical devices have been successfully used in fluorescent immunoassays, offering the prospect of mass-produced miniaturized devices^107,108^.

In electrode manufacturing, graphene has attractive applications in electrochemical sensing due to its fast electron mobility, high current density, and large surface area. Among the most promising two-dimensional (2D) active materials exemplified by graphene, MXene, with its hydrophilic surface and high electrical conductivity, excels in various applications, such as sensors, catalysis, and energy storage. It has been reported that the properties of graphene frameworks can be improved by doping CuxOpolypyrrole conductive aerogels, reducing the weakening due to severe aggregation through strong π–π interactions. This conductive aerogel has been successfully applied to the detection of antibiotics and hydrogen sulfide with excellent performance^109^. Ti_3_C_2_T_*x*_, representative of the MXene family, has a unique 2D morphology, good biocompatibility, and is promising for its application in designing advanced nanohybrid systems for detecting antigen antibodies, proteins, and enzymes^110^. Meanwhile, Ti_3_C_2_ MXene has a high photothermal conversion efficiency and is suitable for photothermal immunoassays^111^, and it can be used to achieve immobilization of biomolecules^112^. Compared with ordinary electrode materials, the silk fabric-derived intrinsically nitrogen (N)-doped carbon textile (SilkNCT) has an inherent hierarchical structure and porous mesh weave structure, which facilitates good contact with reactants and effective electron transfer. With these unique properties, silk-derived carbon fabrics have a promising future as working electrodes for wearable electrochemical sensors^113^.

Methods for fabricating electrodes on flexible substrates include photolithography and printing methods. The screen printing method is a standard sensor manufacturing technique with low cost and is suitable for mass production^114^. Compared to screen, offset, or flexographic printing, the simple printing mechanism of roll-to-roll gravure allows for faster print speeds, higher resolution, and consistency, thus facilitating large-scale, high-throughput manufacturing on flexible substrates of up to 150 m^115^. Lithography, including the use of CO_2_ laser engraving, offers the advantage of high resolution and allows for rapid engraving of patterns in ambient conditions, and reduces personnel training and process optimization^61^. Nevertheless, lithography has the disadvantages of expensive instruments and complicated steps, which limit its wide application.

### Electronic components

Electronic components are a very important part of wearable sweat sensors. The signal generated by the sensing element ultimately needs to be transduced, conditioned, and processed by the electronics to filter out the noise and calibrate and compensate for the signal. Additionally, to facilitate observation, wireless transmission protocols are required for transferring and visualizing the collected information to the terminal device^3,8,21,22^.

For a general wearable sweat sensing device, the main body of electronics consists of an analog front-end to condition the sensor signal, an analog-to-digital converter (ADC), a preprogrammed microcontroller to calibrate the signal to a concentration value, and a wireless communication component to forward this signal to a paired cell phone application for assessment^7^. One of the key roles of electronics is to filter the raw sensor signal to eliminate noise. Motion and interference between various sensing methods are among the sources of noise generation^116^. Analog front ends can use low-pass filters to eliminate high-frequency noise from motion or fluctuations in the sensing layer environment. Some successful sweat sensing platforms utilize hybrid electronics that integrate flexible sensing substrates with traditional silicon integrated circuits for signal processing and transmission. Various topologies and substrates, including soft plastics and polymers with printed conductive elements or flexible PCBs, are available for electronic components^7,17,117–119^.

The choice of wireless communication protocol depends on a series of criteria, such as power consumption, data generation rate, bandwidth requirements, and compatibility with other sensor circuits^120^. Bluetooth low energy (BLE), with a wireless communication range of up to 100 m, has been widely applied in wearable devices. In one example, a fully integrated sensing array was connected to a microcontroller and Bluetooth module for measuring sweat metabolites, electrolytes, and skin temperature^7^. Nevertheless, the high energy consumption of BLE is still unsatisfactory. The near-field communication (NFC) approach allows for signal transmission using the power of the receiving device to trigger sensor measurements and data collection without the need for a battery-power transmission component inside the sensor. NFC is widely deployed in wearable microfluidic devices. For example, NFC coils have been integrated with epidermal microfluidic devices to capture, store and analyze sweat electrolytes in aqueous environments^121^. However, the current problem of NFC is that its wireless communication distance is only 10 cm or less^21^, requires the participation of the users to complete the information transmission, and cannot automatically function continuously. RFID is another wireless communication standard that has been applied to wearable electrochemical sweat sensors^119^. RFID chips allow potential sensing of electrolytes in sweat and skin surface temperature with minimal components and can be used for hydration and thermal stress monitoring.

To facilitate the observation of data, many wearable electrochemical sensing devices have applications that can remotely initiate sensing and record data, which can be viewed on the displays of portable phones and watches^17,73,88,121^. In addition, applications that transform sensor data into useful information that can be mapped to final health or fitness parameters (e.g., hydration levels and fatigue) through calibration curves and feedback control algorithms would be very useful.

### Power components

The support components in wearable devices for data processing and wireless data transmission require power to operate. Moreover, in terms of practicality, wearable devices require the ability to operate continuously for long periods. Therefore, reducing power consumption and developing efficient wearable energy systems are major considerations for wearable devices. Current wearable energy resources fall into two main categories: lithium-ion batteries and energy harvesting devices^122–124^.

Lithium-ion batteries offer the advantages of long operating life and fast recharge. However, it is difficult to make a sufficiently small lithium-ion battery, and thus they do not meet the needs of wearable devices. Efforts have been devoted to making lithium-ion batteries suitable for wearable forms. For instance, a flexible and stretchable battery has been made by placing the cell components of a conventional battery on an elastomeric substrate^123^. However, lithium-ion batteries can also easily explode and cause safety problems. To minimize the risk of overheating lithium-ion batteries expanding and exploding, protective layers have been designed for these batteries that can cause the battery to be powered off in the event of overheating^125^. Zinc-manganese batteries based on quasi-solid water electrolytes eliminate the safety problems of batteries and are lightweight and environmentally friendly and have high output voltage and high capacity, making them one of the most promising alternatives to conventional batteries.

**Fig. 4:**
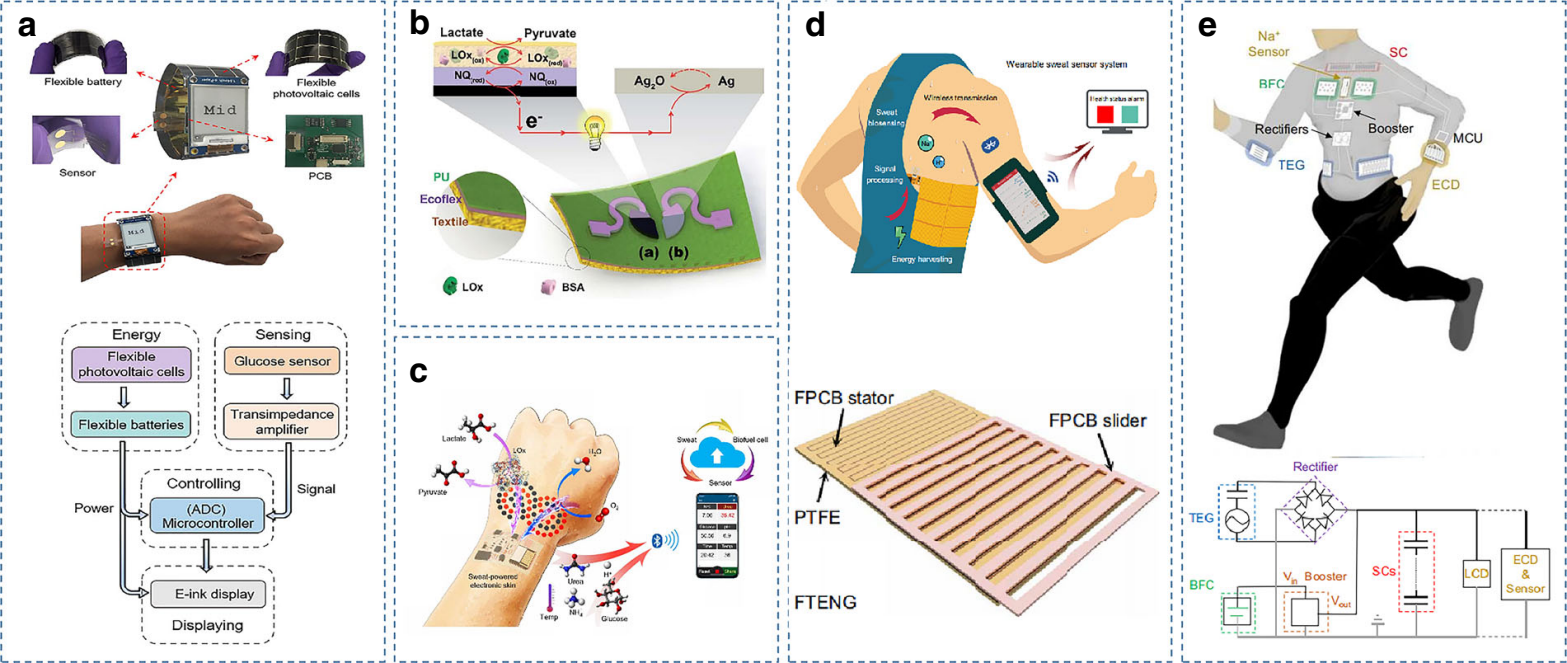
Examples of self-powered sensors. **a** Form factor and system-level block diagram of a self-powered photovoltaic cell-based smartwatch for continuous sweat glucose detection^87^. Copyright 2019, ACS. **b** Working principle of a self-powered wearable sweat sensor based on a biofuel cell^19^. Copyright 2016, RSC. **c** Schematic of a battery-free, biofuel-powered e-skin that efficiently harvests energy from the human body^128^. Copyright 2020, AAAS. **d** Schematic diagram of a triboelectric nanogenerator-based motion-driven wearable sweat sensor^130^. Copyright 2020, AAAS. **e** System diagram of the energy microgrid system, consisting of the TEG, BFC, and SC modules, and the wearable applications^20^. Copyright 2021, Springer Nature

Meanwhile, to meet the growing power demand of wearable electronic devices and to avoid frequent interruptions in charging and cumbersome wired power transmission, wearable systems integrate energy harvesting devices such as solar cells, triboelectric nanogenerators (TENGs), and microbial biofuel cells (BFCs) to achieve self-sustainable operation^32^. Through flexible photovoltaic cells that can collect solar energy and convert it to the energy required by sensors, there is a stable self-powering capability and high energy storage capacity to support the continuous functionality of the system. Figure 4a shows a smartwatch that uses flexible photovoltaic cells for energy collection/conversion and flexible zinc-manganese batteries as energy storage devices to continuously monitor sweat glucose levels without external charging facilities^93^. The sensor exhibits stable self-power with a high energy storage capacity to support the continuous functionality of the system.

BFCs can convert molecules such as glucose, lactate, uric acid, and ethanol present in biofluids into energy to power sensors and implanted devices, including pacemakers, pumps, and stimulation electrodes. Human sweat can be used as an ideal sustainable bioenergy source to power wearable sweat sensors^126^. In 2018, the first wearable and scalable self-powered biosensor were reported^19^. As shown in Fig. 4b, a highly stretchable textile-based BFC was fabricated by utilizing a screen printing process based on stretchable custom inks and a custom stencil and electrode design, allowing the extraction of electrical energy from sweat for the detection of lactate. The great potential of self-powered sensing and energy harvesting operations in the field of wearable sensors has been fully demonstrated. However, epidermal BFCs can only generate and transfer energy in the presence of sweat. Different levels of sweat lactate BFCs can limit the constant power output due to irregular sweating. In addition, the limited BFC output voltage requires additional electronic components, which limits the flexibility of the overall device. Supercapacitors may provide the solution. Using a sweat-based BFC to derive energy from lactic acid and then storing it by an integrated supercapacitor (SC) can provide stable energy output over long periods and maintain good rechargeability^127^. Due to their excellent performance, lactate fuel cells have recently been successfully integrated into multimodal wearable sweat sensors. As shown in Fig. 4c, a perspiration-powered integrated electronic skin (PPES), which can be fully driven by sweat, uses a unique zero- to three-dimensional nanomaterial integration that further improves the power strength and long-term stability of the BFC, enabling selective continuous monitoring of key metabolic analytes (urea, NH4^+^, glucose, and pH) in sweat^128^.

TENGs convert mechanical energy generated by human movement into electrical energy through the coupling of induction and frictional electric effects^129^. Since they operate independently of uncontrollable external sources such as sunlight or wireless power transmitters, they can power wearable sweat sensors during intensive physical activity. For example, as shown in Fig. 4d, a wearable freestanding-mode TENG (FTENG) converts the mechanical energy generated during human movement into electrical energy and integrates efficient power management integrated circuit (PMIC) to store and release the energy. Energy can be efficiently harvested from human skin and continuously powered for wearable devices^130^.

As a high-capacity capacitor that stores much more energy per unit volume than available batteries or electrolytic capacitors, ultracapacitors can withstand more charge/discharge cycles and receive or transfer charge at a faster rate. The energy harvesting device collects energy from the environment and then conditions and stores the cleared energy in the supercapacitor, which then supplies the stored energy to the wearable sensor as needed^131^.

In hybrid harvesting systems with multiple energy input modes, system-level considerations are needed to guide the wise selection of components with complementary characteristics and commensurate performance to efficiently and reliably remove energy from human activities^132^. The microgrid is a microscale grid with components for power generation, energy storage, various utilities, and management functions for regulating energy flow^133^. Such self-sustainable microgrids can operate independently of the main grid, harvesting energy from local sources and regulating and storing the removed energy in various energy storage modules. As shown in Fig. 4e, energy is harvested from sweat and from exercise using sweat-based biofuel cells and friction nanogenerators, while the collected energy is stored and regulated by supercapacitors. The device utilizes limited energy to provide autonomous and sustainable power for wearable devices^20^.

### Typical wearable electrochemical sweat sensors

Sweat is rich in electrolytes, metabolites, trace elements, and various small molecules, which provide potential insights into different aspects of human metabolic and physiological states. Wearable electrochemical sweat sensing devices, which have been extensively studied recently, provide unprecedented real-time access to monitor analytes in sweat, offering a powerful method for health monitoring and disease detection. Meanwhile, the choice of electrochemical detection technique depends largely on the target analyte. In this section, we will introduce some typical wearable sweat sensors based on different analyte types and significant milestones in technological development.

### Electrolyte and metabolite monitoring

In the evolution of wearable sweat sensors, the detection of electrolytes and metabolites is the most developed and widely applied, providing information on body hydration, osmotic balance, muscle fatigue, blood glucose levels, etc. For the detection of electrolytes in sweat, generally ion-selective electrodes (ISEs) and potentiometric methods are employed. Conventional ion-selective sensors consist of a membrane-based ion-selective electrode and a reference electrode, both of which require an internal solution to ensure a stable and sensitive response^92^. Its advantages include low sample volume requirements, miniaturization, cost reduction, versatility, and simplicity of in situ sensing of biological materials^134^. The assay utilizing enzymatic reaction and amperometry is well suited for the detection of a range of metabolites. These analytes readily undergo redox reactions in the presence of selective enzymes. The concentration of the analytes can be detected by monitoring the current generated by the oxidation or reduction reaction between electroactive substances at a constant potential. This method offers the advantages of simplicity of operation, provides a fast and continuous response, and minimizes the lag time between noninvasive wearable measurements and blood analyte concentrations.

**Fig. 5:**
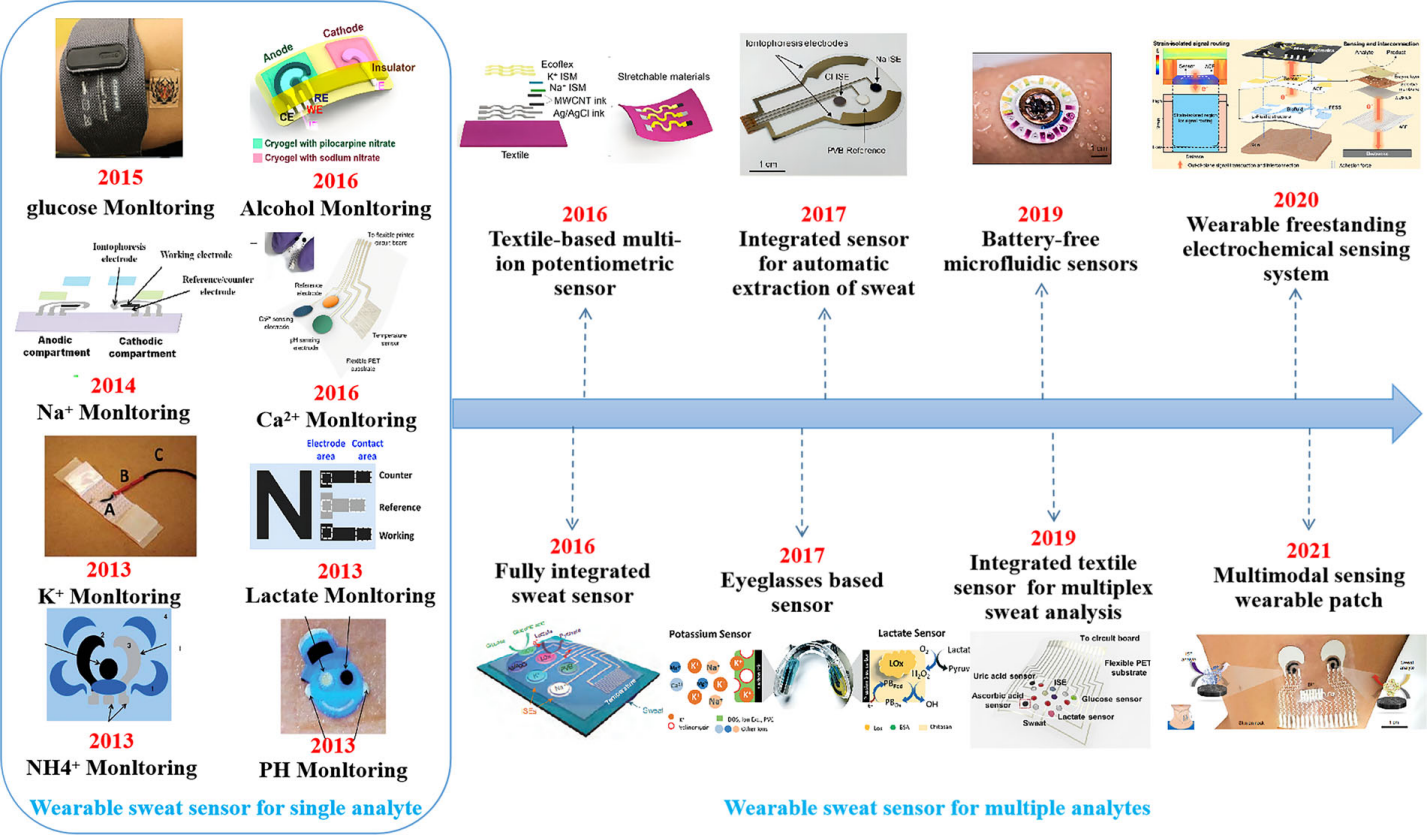
History of wearable electrochemical sweat sensors for detecting metabolites and electrolytes. In 2013, the first wearable electrochemical sweat sensor was developed for real-time noninvasive sweat lactate monitoring^11^. Subsequently, several wearable sweat potentiometric sensors were developed, employing ion-selective electrodes and enabling noninvasive monitoring of pH^86^, NH_4_^+^^55^, K^+^^140^, Na^+^^42^, and Ca^2+,^^54^ in sweat. Wearable sweat sensors for metabolite monitoring, such as glucose^131^ and alcohol^16^, have also been developed. 2016 saw milestone advances in wearable sweat sensors. For example, a wearable sweat sensor for multiplexed biosensing, allowing simultaneous detection of multiple analytes in sweat, with integrated circuits for signal processing and wireless data transmission, was developed^7^. Copyright 2016, Springer Nature. At the same time, a textile-based multi-ion sweat analysis potentiometric sensor with high stretchability was developed^53^. Copyright 2016, Wiley. In 2017, a wearable sweat sensor that automatically extracts and analyzes sweat was reported, designing an electrochemically enhanced iontophoresis interface, allowing simultaneous detection of chloride and glucose in sweat^17^. Copyright 2017, National Academy of Sciences USA. Moreover, a commercially available glasses-based electrochemical sweat sensor was developed, which detects both potassium and lactate in sweat^52^. Copyright 2017, RCS. In 2019, a battery-free microfluidic wearable sweat sensor based on lactate biofuel cells was developed^133^. Copyright 2019, AAAS. An integrated textile sensor patch for real-time and multiplex sweat analysis was reported, improving sensitivity and selectivity^109^. Copyright 2019, AAAS. In 2020, a wearable electrochemical sensing system was developed that integrates sweat sampling, electrochemical sensing, and data display/transmission on the same platform^135^. Copyright 2020, AAAS. Recently, in 2021, a multimodal wearable biosensor was developed to perform continuous, simultaneous acoustic and electrochemical sensing^134^. Copyright 2021, Springer Nature

The history of wearable electrochemical sweat sensors for detecting metabolites and electrolytes is shown in Fig. 5. Early research on wearable sweat sensing platforms focused on single analyte sensing of a wide range of target analytes. In 2013, Joseph Wang’s group developed the first flexible wearable tattoo-based electrochemical sweat sensor for real-time noninvasive detection of lactate in human sweat^11^. The sensor used a lactate oxidase (LO*x*)-modified sensing electrode to detect sweat lactate concentration by the chrono-current method with a high degree of selectivity. In addition, a traditional screen printing method was employed to create the transfer tattoo. This enabled the sensor to withstand the repetitive mechanical deformation that the epidermis undergoes during movement, and the sensor can be easily worn by a person. In a subsequent study, the detection range of the sweat epidermal tattoo sensor was extended, and the sensor included glucose monitoring^135^.

Meanwhile, pioneering results have been achieved in electrolyte detection. A new tattoo-based ion-selective sensor was reported^92^. A polyaniline (PANI)-modified ion-selective electrode was used as the pH-sensitive electrode, making the device selective and biocompatible. Combining techniques such as commercial temporary tattoo paper with conventional screen printing and solid contact polymer ISE methods, a potentiometric-type electrochemical detection method was used to enable rapid, real-time monitoring of pH levels in human sweat during exercise. Subsequently, noninvasive real-time monitoring of electrolytes such as ammonium ions (NH4^+^)^55^, potassium ions (K^+^)^136^, and calcium ions (Ca^2+^)^54^ in sweat was achieved. Furthermore, polyvinyl butyral (PVB) was employed for the first time as a solid-state reference membrane in a wearable device.

Wires and connectors for data transmission greatly interfere with human activity. An epidermal tattoo potential sensor with the wireless signal transmission was developed in 2014, incorporating several state-of-the-art thick film technologies, laser printing technology, solid-state potential measurement technology, fluid technology, and wireless technology. Real-time sodium concentration in sweat can be transmitted to a laptop 10 m away via a wireless Bluetooth transceiver^42^. Soon after, Hickenfeld’s group reported the first wearable sensor that transmitted wirelessly to a user interface, achieving a breakthrough in delivering detected information to the user^119^. The sensing platform is based on a radio frequency identification (RFID) adhesive patch that potentially senses electrolytes in sweat as well as skin surface temperature, allowing data to be transferred to a smartphone for analysis and monitoring hydration in sweat.

However, all of these previously reported wearable sweat sensing platforms suffer from some drawbacks. Because only a single analyte can be monitored at a time, it is not possible to elucidate whether the sensor displays signal changes due to changes in analyte concentration, sweating rate, or sensor malfunction. In addition, these sensing platforms lack in situ signal processing circuitry and sensor calibration mechanisms to accurately analyze physiological states. In 2016, Javey’s group demonstrated a milestone development by demonstrating the first fully integrated, multiplexed flexible integrated sensing array (FISA) that selectively detects electrolytes (sodium, potassium) and metabolites (glucose, lactate) in sweat and measures skin temperature simultaneously^7^. The sensing component consists of an enzyme-based biosensing current sensor that detects glucose and lactate and an ion-selective electrode (ISE)-based potential sensor that detects sodium and potassium, enabling continuous multiplexed measurements. The signal conditioning, processing, and control circuitry of the integrated circuit assembly and the Bluetooth wireless transmission module are also integrated using FPCB technology to calibrate the raw analyte signals to meaningful concentrations and transmit data via Bluetooth transceiver to a customized cell phone application for data analysis. The sensing platform integrates plastic-based sensors and traditional integrated circuit assemblies at an unprecedented level of integration, revealing an extremely promising future for wearable electrochemical sweat sensors. Recently, an integrated wearable electrochemical sweat analysis device was reported that simultaneously detects six health-related biomarkers in sweat^113^. By adopting silk fabric-derived intrinsically nitrogen (N)-doped carbon textile (SilkNCT) with high conductivity as the working electrode, the previous wearable electrochemical sweat sensor with low sensitivity and selectivity in measuring multiple analytes simultaneously was successfully surpassed.

These methods, however, involve a complex collection of hardware and are difficult to miniaturize for the daily convenience of the wearer. A new electrochemical sensing approach is proposed, similar to a biofuel cell, which does not require additional instruments, and the target analytes spontaneously generate electrical signals proportional to their concentration^137^. Biofuel cell-based electrochemical sensors for glucose and lactate and a device for colorimetric analysis of chloride are integrated on a microfluidic substrate, allowing for sweat sample processing with zero crosstalk between different sensors. Small, low-power, battery-free NFC electronics were selected for wireless data transmission, enabling simultaneous monitoring of sweating rate, sweat volume, pH, lactate, glucose, and chloride.

Sensor integration is now shifting to the combination of different sensor modalities, and there have been breakthroughs in the combination of sweat sensors with different biofluid sensors (e.g., interstitial fluid sensors) and physical sensors. Recently, a highly integrated multimodal flexible and scalable wearable sensor has been reported, which overcomes the engineering challenges associated with different sensing modes and materials; it uses ultrasonic sensors to monitor human blood pressure and heart rate and electrochemical sensors to achieve comprehensive and dynamic self-monitoring of human health status by using lactic acid, alcohol, and caffeine in sweat and glucose in interstitial fluid (ISF)^138^.

In addition to traditional epidermal wearable sweat sensors such as patches and tattoos, other platform approaches have gained significant expansion. Joseph Wang’s group reported the first fully integrated glasses-based wireless multiplexed sweat sensing platform in 2017^52^. The sensing platform integrates amperometry for detecting sweat lactate and a potential sensor for detecting potassium in sweat on each nose bridge pad of the eyewear. It also integrates an electronic backbone in the eyewear frame to provide control of the current and potential sensors and supports Bluetooth wireless data transmission to the host device. This new eyewear platform offers better comfort and suitability for the wearer and meets the user’s need for esthetics with great commercial potential. A 2020 report demonstrated a smartwatch supporting a stand-alone sensor system with sweat sampling, electrochemical sensing, and data display/transmission in sedentary and high-intensity exercise situations. In addition, by examining the biomarker messaging pathway and identifying near-zero strain regions within the microfluidic-based sensing module, the strain isolation pathway was designed to maintain high fidelity of biomarker data, which has great potential for large-scale clinical applications^139^.

### Heavy metal and drug detection

**Fig. 6:**
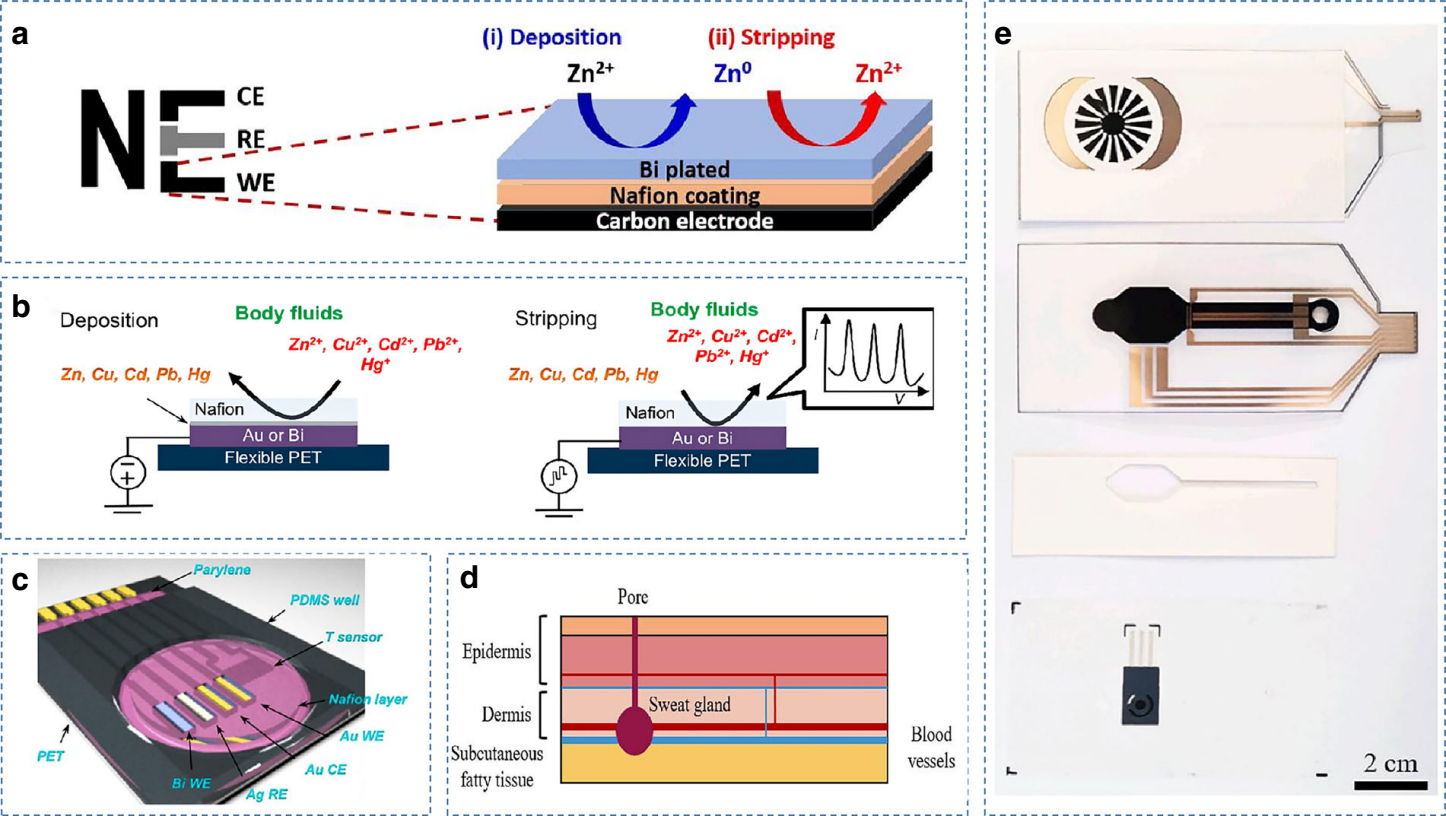
Examples of sweat sensors for heavy metal detection. **a** Schematic of a wearable sweat sensor for real-time detection of trace amounts of metallic zinc in sweat^67^. Copyright 2015, Elsevier. **b** and **c** Schematic of a wearable sensor for multiplexed heavy metal monitoring of sweat^66^. Copyright 2016, ACS. **d** and **e** A wearable and fully printed microfluidic nanosensor for copper detection in sweat^68^. Copyright 2022, Elsevier

Human sweat contains a variety of trace elements that are closely related to the health status of the body. In 2015, Joseph Wang’s group developed the first example of a wearable electrochemical trace metal sensor by applying a bismuth/sodium fluoride-coated electrode with excellent properties for trace metal detection. The sensor uses square wave anodic stripping voltammetry (SWASV) to achieve real-time monitoring of zinc in human sweat (Fig. 6a)^67^. Subsequently, Gao et al. developed a wearable flexible sensing array using the same electrochemical detection method, calibrated and compensated with a skin temperature sensor (Fig. 6b). Multiplexed simultaneous selective detection of heavy metal elements such as zinc, cadmium, lead, copper, and mercury ions in sweat was achieved^66^. A fully printed, wearable microfluidic nanosensor has recently been reported that integrates a reverse iontophoresis system and a wireless device for continuous, real-time detection and quantification of copper secreted in sweat (Fig. 6d)^68^.

**Fig. 7:**
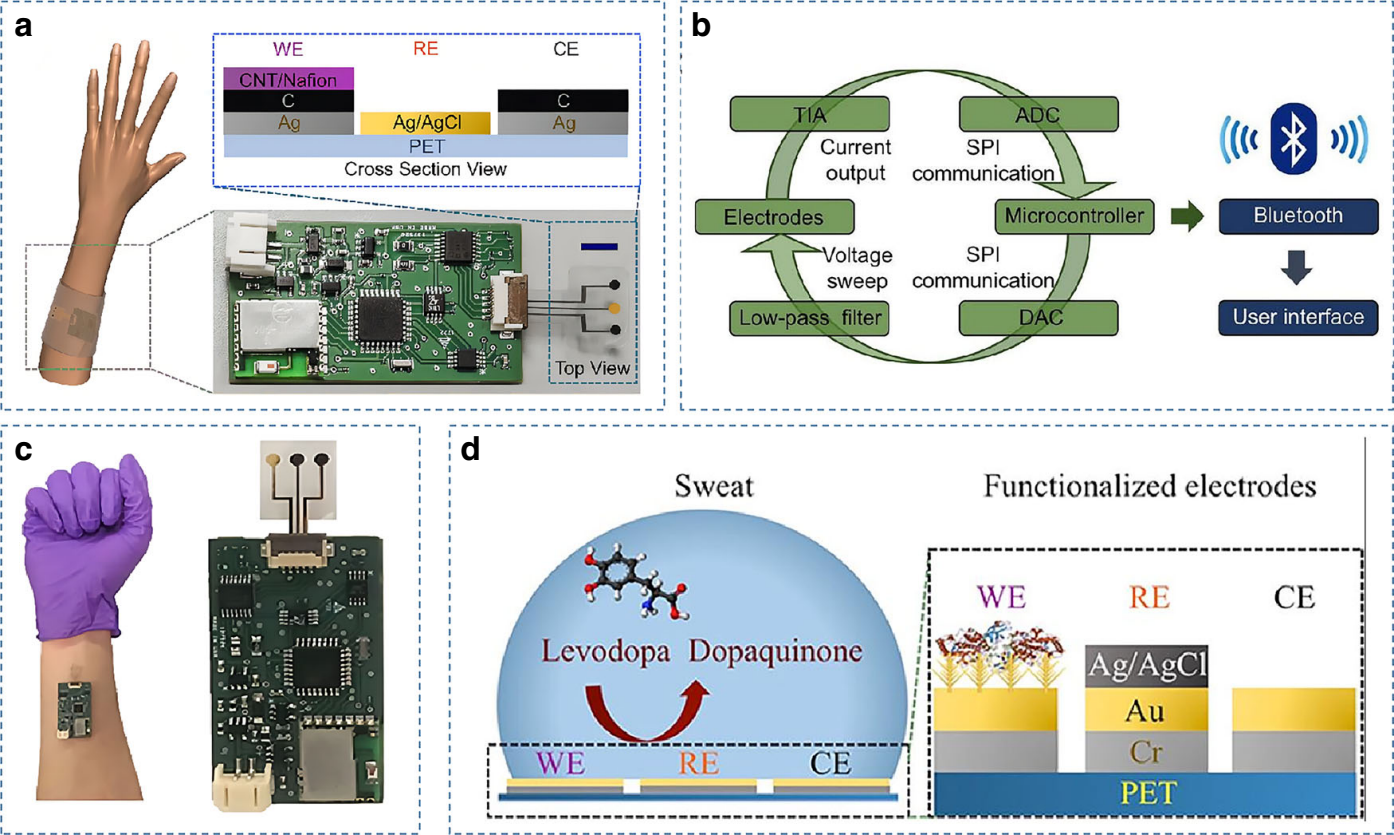
Example of a wearable sweat sensor for drug detection. **a** and **b** Schematic and system-level diagram of a wearable sweat sensor for caffeine drug monitoring^63^. Copyright 2018, Wiley. **c** and **d** Schematic of a wearable sweatband for noninvasive levodopa monitoring and its sensing mechanism^64^. Copyright 2019, ACS

Drug monitoring plays a crucial role for physicians to accurately adjust drug doses and understand the complex pharmacokinetics of drugs. However, in wearable sweat sensing assays, the detection of drug molecules is challenging due to their extremely low concentration in biofluids. A wearable sensing platform used for drug detection in sweat was first reported in 2018^63^ (Fig. 7a). Differential pulse voltammetry (DPV), based on the oxidation of the target molecule at its unique potential and then measuring the corresponding current, was used to allow sensitive determination of the concentration of the measured molecule in sweat. Roll-to-roll printing technology is utilized to enable the mass production of high-performance electrode arrays. At the same time, integrated signal transduction, modulation, processing, and Bluetooth transmission functions allow for noninvasive, real-time, and in situ monitoring of methylxanthine drugs (caffeine) in sweat. This groundbreaking drug detection work greatly facilitates pharmacokinetic studies. Figure 7c shows another breakthrough in drug detection. By adding gold dendritic nanostructures to significantly improve the stability of the electrode, noninvasive detection of levodopa in sweat was successfully achieved^64^.

### Other target detection

Sweat has a wealth of other components, including cytokines, hormones, and proteins. By detecting these molecules in sweat, a deeper understanding of body homeostatic mechanisms and overall body health can be obtained. However, there have been few reports of continuous monitoring of small molecule biomarkers in a sweat over a long duration. The main difficulty is that these analytes change in composition and cause different changes in pH over time and at low concentrations, making it difficult to achieve stable detection using electrochemical sweat sensors^140^. Detection can be performed using antibodies or aptamers for affinity-based sensing^22^, as well as molecularly imprinted polymer (MIP)-based methods^72^. For this class of analytes, electrochemical impedance spectroscopy (EIS) can be used to probe the affinity status of the electrode surface by analyzing the complex impedance in the Nyquist diagram. In addition, cyclic voltammetry (CV) can also be used for detection.

**Fig. 8:**
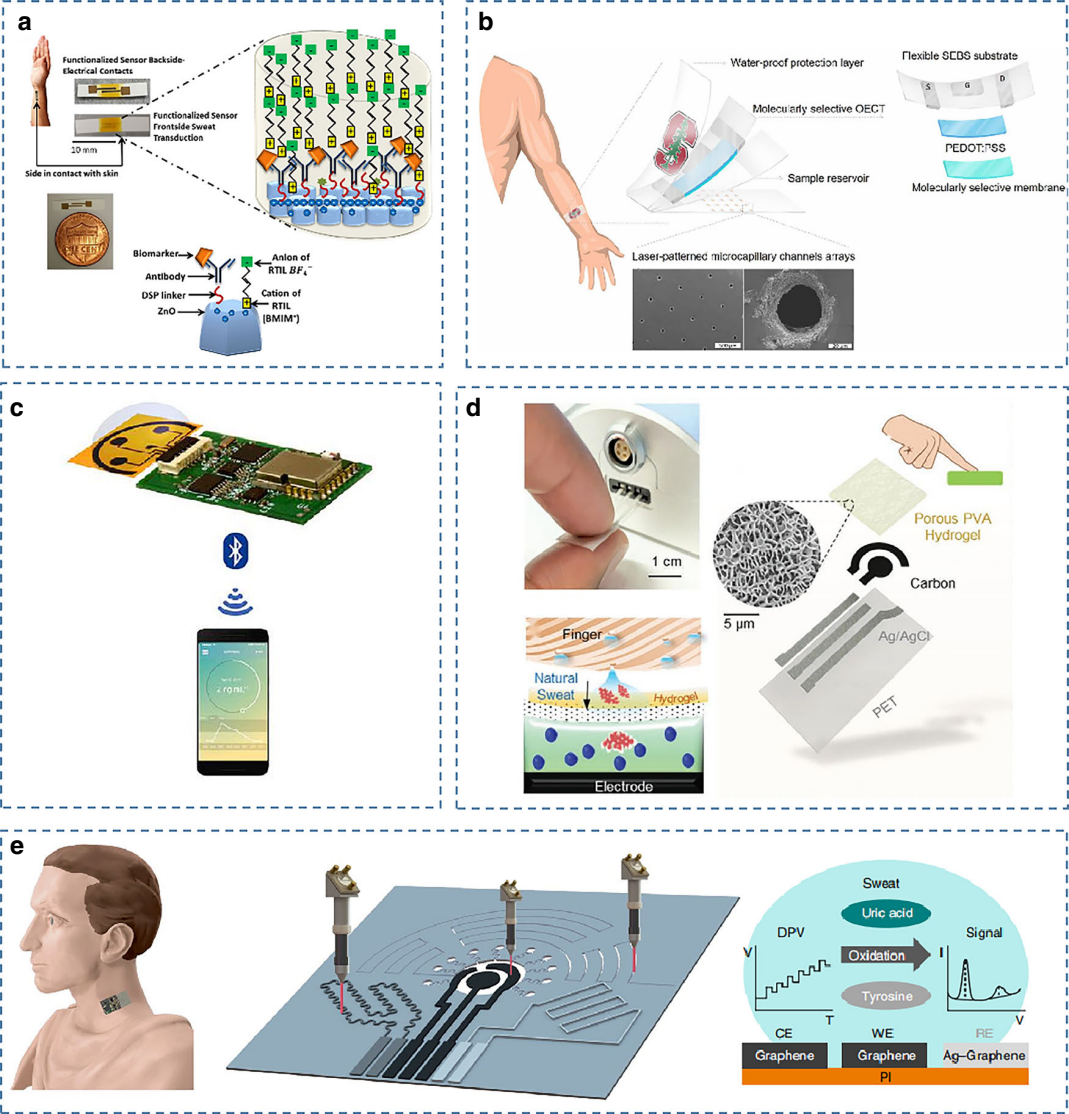
Examples of wearable sweat sensors for monitoring other analytes. **a** A wearable sweat sensor based on room-temperature ionic liquids, enabling the detection of interleukin 6 and cortisol in sweat^69^. Copyright 2017, AAAS. **b** A wearable organic electrochemical noninvasive cortisol-sensing device based on molecularly selective nanoporous membranes^73^. Copyright 2018, AAAS. **c** A graphene-based wearable sweat sensor for sweat cortisol detection^59^. Copyright 2020, Elsevier. **d** A fast, stress-free MIP-based cortisol sensing sensor^72^. Copyright 2021, Wiley. **e** A wearable laser-engraved sensor for the sensitive detection of uric acid and tyrosine in sweat^61^. Copyright 2020, Springer Nature

In 2017, Prasadt’s group reported a wearable sweat sensor based on functionalized antibodies^69^. As shown in Fig. 8a, nanoporous polyamide membranes are used as substrates to fabricate sensing arrays, and capture probes (antibodies) are immobilized in room temperature ionic liquid (RTIL) to improve the stability of antibodies. The sensing platform enables the first combined detection of IL-6 and cortisol in human sweat. The high stability of the sensor performance was also verified using electrochemical impedance spectroscopy (EIS), ATR-IR spectroscopy, and dynamic light scattering (DLS) techniques.

Graphene, with its large surface area and fast electron mobility properties, has superior performance in electrochemical sensing. Competitive immunosensing strategies provide significant advances in the detection of highly selective small hormone molecules. A wireless sensing device combining laser-induced graphene and a competitive immunosensing strategy was developed for rapid, reliable, sensitive, and noninvasive monitoring of the stress hormone cortisol (Fig. 8c). As a result of the study, the first cortisol circadian cycle and dynamic stress response curves constructed from human sweat were reported, and a strong empirical correlation between serum and sweat cortisol was verified^59^. Thread-based electrochemical immunosensors were recently reported for the first time, enabling the noninvasive detection of cortisol in sweat by immobilizing cortisol antibodies on l-cys/AuNPs/Mxene-modified conductive thread electrodes, significantly improving the sensitivity of the sensors^141^.

Although competitive immunosensing approaches offer high sensitivity, their complex sensing procedures make it difficult to meet the needs of wearable applications. MIPs are used to mimic enzyme–substrate or antibody–antigen interactions for the specific recognition of blotted molecules, with a range of properties, such as high selectivity, sensitivity, reliability, low cost, and mechanical, thermal, and chemical stability^142^. Figure 8b shows an organic electrochemical transistor (OECT), which adopts an artificial recognition membrane based on molecularly imprinted polymers (MIPs) as a specific recognition element. The limitations of OECTs, which are not suitable for the detection of cortisol due to their uncharged nature at physiological pH, were overcome. This sensing platform allows stable and selective molecular recognition of cortisol in sweat^73^. Nevertheless, such MIP-based sensing usually requires prolonged physical activity or additional iontophoretic stimulation of sweat glands to obtain sweat, which may increase the user’s psychological stress and thus alter cortisol levels, leading to inaccurate stress assessment. Figure 8d shows a wearable touch-based cortisol sensor described by Tang et al. The use of a highly permeable perspiration-wicking porous polyvinyl alcohol (PVA) hydrogel allows for fast and effective sweat collection. Molecularly imprinted polymer (MIP) electrochemical sensor technology was used to achieve fast, simple, and reliable detection of sweat cortisol through a highly selective combination with a cortisol-imprinted electropolymerized polypyrrole coating^72^. The sensing platform has been experimentally validated to have significant advantages over common cortisol measurements in capturing cortisol levels during sharp fluctuations. This MIP-based fingertip cortisol sensing provides a reliable and practical method for rapid and stress-free pressure monitoring.

Previously, the measurement of uric acid and tyrosine in sweat was challenging due to their low concentrations. Figure 8e shows a laser engraved sensor. Utilizing the advantages of graphene in electrochemical sensing, rapid and accurate in situ detection of uric acid and tyrosine in human sweat was achieved by using differential pulse voltammetry (DPV) to assess ultralow levels of uric acid and tyrosine based on the amplitude of the peak oxidation current^61^.

## Conclusion and outlook

Sweat plays an important role in health monitoring and disease detection as an ideal bioassay sample that contains rich biomarkers that can indicate physiological information about the human body. After years of development, great progress has been made in flexible wearable electrochemical sweat sensors. They show promise for the detection of analytes such as electrolytes, metabolites, trace metals, drugs, and small molecule compounds. Research on skin interface microfluidics, flexible stretchable materials, self-powered technologies, and multiplexed sensing modes are driving the development of wearable electrochemical sweat sensors. However, many aspects of wearable electrochemical sweat sensing must be further developed to advance the field and enable personalized, intelligent medicine:

### Toward integrative, multifunctional perspiration analysis

Great progress has been made in the integration of wearable electrochemical sensors. The integration of a wireless data transmission device on the electrochemical sensor and the development of a smartphone application for visualizing and analyzing data has made it possible for users to easily view physiological information and improve the usefulness of the sensing device. Merging electrochemical sensors with silicon integrated circuit assemblies with complex electronic functions allows an accurate assessment of the human physiological state to be obtained by reducing the effect of signal noise through signal processing. In terms of sweat stimulation and collection, the integration of an iontophoresis device makes it possible to obtain sweat by chemical stimulation while the body is at rest. The incorporation of microfluidics makes the collection and analysis of sweat faster and more efficient, allowing the sensor to detect the analytes at low concentrations. In parallel, sensors for the simultaneous detection of multimodal analytes in sweat are rapidly evolving. While most early wearable devices focused primarily on individual measurements, there has been a shift to simultaneous noninvasive monitoring of a broad range of biomarkers. This more comprehensive analysis not only allows for a broader analysis of physiological states but also allows for active calibration and correction of responses for more accurate monitoring. In addition, sensor integration is now shifting to a combination of different sensor modalities. Sensors that detect different biofluids can be combined, such as simultaneous detection of lactate in sweat and glucose in ISF. It is even possible to integrate biosensors with physical sensors to achieve more comprehensive monitoring of human physiological information.

In the future, sensors will undoubtedly be more integrated and intelligent. On the one hand, more functions can be integrated into the sensing device. We envision that advanced big data processing algorithm modules based on machine learning or deep learning will be integrated into the sensors to achieve fast and accurate analysis of the collected data. On the other hand, multimodal wearable sensors that integrate chemical, electrophysiological and physical sensors can be further developed to integrate multiple sensing modalities into a single sensing platform, comprehensively monitoring human physiological information.

### Improvement of the reliability of sweat samples

Several key challenges have yet to be addressed for the successful realization of reliable and accurate real-time monitoring in sweat. First, sweat sensors need to simultaneously monitor sweating rate, identify and compensate for sweating rate effects, and provide a comprehensive understanding of biomarker distribution mechanisms and their dependence on sweating rate. The sweat secretion rate varies depending on the individual or the environment. For example, Na^+^ and Cl^−^ are usually more concentrated at higher sweating rates. The ideal wearable sensor must have good adaptability to meet the need for detection at different sweating rates.

Second, wearable sweat sensors should ideally be able to select different sweat sample collection methods depending on the needs. The composition of sweat samples obtained using different methods may be different. Exercise-induced sweat may contain more metabolites, such as lactate, than sweat collected after iontophoretic stimulation.

Third, measures should be taken to minimize changes in sweat composition during the testing process. Small amounts of exposed sweat evaporate rapidly, thus changing the concentration of constituent biomarkers in the sweat sample. Additionally, fresh sweat secreted onto the skin surface mixes with old sweat. Without technology to control sweat flow so that detection occurs only in the freshest sweat, the sensor cannot achieve real-time measurement of sweat.

Finally, the contamination of sweat samples is an urgent issue that needs to be addressed. Contaminants on the skin can be mixed with the sweat produced, thus altering the composition of the sweat in the collected sample. Isolating the sweat from the skin surface is critical to prevent these interfering chemicals from affecting the sensor readings.

### Development of efficient energy utilization devices

Due to the integration of electronic components such as those facilitating wireless data communication, the power consumption of wearable electrochemical sweat sensors is a serious problem. To achieve continuous and stable monitoring of analytes in sweat, the best method of powering these devices needs to be explored. Traditional lithium-ion batteries are heavy and bulky and cannot adequately meet the user’s need for wearability. Flexible and stretchable energy storage batteries have been developed to provide stable performance under varying degrees of mechanical deformation. However, their mass adaptation is inconveniently limited by the manufacturing process, low energy density, and frequent recharging. Integrating energy-harvesting devices and finding sustainable alternative energy sources is a promising approach. Energy harvesting from solar energy, body movement, and human biofluids has already been successfully implemented. Direct integration of these devices with energy storage devices allows the creation of self-powered wearable electrochemical sweat sensing systems. Nevertheless, current technologies are far from meeting the requirements of providing stable and reliable energy support for existing high-performance wearable bioelectronic devices that perform multiple complex tasks. Integrating multiple energy harvesting devices into the same platform and using efficient energy storage and control would help to improve the energy supply. At the same time, in addition to finding alternative energy sources, some energy-saving solutions can be investigated. For instance, deep learning or artificial intelligence modules can be employed to understand when and how to efficiently collect data from various electrochemical or electronic sensors.

### For the detection of low concentration analytes in sweat

Sweat contains a wealth of other components, including hormones, proteins, and peptides. The detection of these molecules in sweat can provide deeper physiological insight into the body’s homeostatic mechanisms and the overall state of health of the body. However, the concentrations of these analytes are low and can be altered by factors including pH and temperature. Hence, determining these components in sweat is challenging. There is a need for highly sensitive wearable sensors that can monitor low concentrations of analytes in sweat through effective sensing patterns. By effectively controlling sweat within the microfluidic channel, the microfluidic device allows for rapid and accurate detection of analytes at low concentration conditions. MIP-based electrochemical sensors are designed to achieve sensitive monitoring through the highly selective binding of target analytes. The detection of cortisol in sweat has been achieved and the same techniques can be extended to detect other hormones and biomarkers in the future. Refinement of measurement techniques and sensor platforms for these molecules will enable the expansion of detection capabilities to a new class of physiologically relevant analytes, providing additional options for gaining insight into the physiological health status of the human body.

### Methods of improvement for sweat stimulation and collection in sedentary environments

Developing methods to induce sweat secretion in a sedentary environment is critical for applications such as disease detection. Iontophoresis has shown promise, but the approach cannot be used for continuous monitoring because of the damage caused to the skin by prolonged current cautery. Further development of highly miniaturized sensors with reduced current density requirements that can operate at low secretion rates is needed. This could also be addressed by developing alternative technologies for biomarker extraction.

In addition, the natural sweating of the body at rest or in thermally stimulated environments may provide unique insights into body physiology. The rate of resting sweat secretion may reflect sympathetic nervous system activity induced by an underlying health condition. Increased or suppressed sweating at rest would further indicate autonomic dysfunction, diabetes, cerebrovascular disease, Parkinson’s disease, chronic psychological stress, anxiety, or pain. However, the rate of sweat secretion when a person is at rest is extremely low, and most wearable electrochemical biosensors are inadequate for its monitoring. Improved microfluidic devices utilizing hydrophilic materials may address this problem; more effective solutions need to be developed in the future.
